# Differential Expression of Acid – Sensing Ion Channels in Mouse Primary Afferents in Naïve and Injured Conditions

**DOI:** 10.3389/fncel.2020.00103

**Published:** 2020-05-19

**Authors:** Melina Papalampropoulou-Tsiridou, Simon Labrecque, Antoine G. Godin, Yves De Koninck, Feng Wang

**Affiliations:** ^1^CERVO Brain Research Centre, Québec Mental Health Institute, Québec, QC, Canada; ^2^Graduate Program in Neuroscience, Université Laval, Québec, QC, Canada; ^3^Department of Psychiatry and Neuroscience, Université Laval, Québec, QC, Canada

**Keywords:** dorsal root ganglia, peripheral nerve injury, *in situ* hybridization, neuropathic pain, peptidergic afferents

## Abstract

Injury and inflammation cause tissue acidosis, which is a common feature of various painful conditions. Acid-Sensing Ion channels (ASICs) are amongst the main excitatory channels activated by extracellular protons and expressed in the nervous system. Six transcripts of ASIC subunits including ASIC1a, ASIC1b, ASIC2a, ASIC2b, ASIC3, and ASIC4 are encoded by four genes (Asic1–4) and have been identified in rodents. Most ASIC subunits are present at substantial levels in primary sensory neurons of dorsal root ganglia (DRG) except for ASIC4. However, their expression pattern in DRG neurons remains largely unclear, mainly due to the lack of antibodies with appropriate specificity. In this study, we examined in detail the expression pattern of ASIC1-3 subunits, including splice variants, in different populations of DRG neurons in adult mice using an *in situ* hybridization technique (RNAscope) with high sensitivity and specificity. We found that in naïve condition, all five subunits examined were expressed in the majority of myelinated, NF200-immunoreactive, DRG neurons (NF200^+^). However, ASIC subunits showed a very different expression pattern among non-myelinated DRG neuronal subpopulations: ASIC1 and ASIC3 were only expressed in CGRP-immunoreactive neurons (CGRP^+^), ASIC2a was mostly expressed in the majority of IB4-binding neurons (IB4^+^), while ASIC2b was expressed in almost all non-myelinated DRG neurons. We also found that at least half of sensory neurons expressed multiple types of ASIC subunits, indicating prevalence of heteromeric channels. In mice with peripheral nerve injury, the expression level of ASIC1a and ASIC1b in L4 DRG and ASIC3 in L5 DRG were altered in CGRP^+^ neurons, but not in IB4^+^ neurons. Furthermore, the pattern of change varied among DRGs depending on their segmental level, which pointed to differential regulatory mechanisms between afferent types and anatomical location. The distinct expression pattern of ASIC transcripts in naïve condition, and the differential regulation of ASIC subunits after peripheral nerve injury, suggest that ASIC subunits are involved in separate sensory modalities.

## Introduction

Tissue injury and inflammation heighten pain sensitivity to mechanical, thermal and chemical stimuli through peripheral and central mechanisms (Baron et al., 2010; Pinho-Ribeiro et al., 2017). At the site of injury or inflammation, protons are amongst the first components that are released, leading to local pH decrease and extracellular acidosis, which depolarizes nociceptive free nerve endings in the periphery and induces pain (Issberner et al., 1996; Baumann et al., 2004).

Both Acid-Sensing Ion Channels (ASICs) and Transient Receptor Potential V1 (TRPV1) channels can be activated by protons and are amongst the main sensors for extracellular acidosis in the nervous system (Lingueglia, 2007; Sugiura et al., 2007). Nevertheless, ASICs have higher pH sensitivity (Wemmie et al., 2013) than TRPV1 channels which are activated with pH below 6.0 (Alawi and Keeble, 2010) making ASICs better candidates to sense small pH variations and respond to moderate acidification conditions.

ASICs are members of the degenerin–epithelial sodium (DEG–ENaC) channel family (Waldmann et al., 1996; García-Añoveros et al., 1997; Waldmann et al., 1997) and are directly gated by extracellular protons. Functional ASIC channels are trimeric and composed of homologous or heterologous subunits (Jasti et al., 2007). Four genes (Asic1-4), encoding six different subunits (ASIC1a, ASIC1b, ASIC2a, ASIC2b, ASIC3, and ASIC4) through alternative splicing, have been identified in rodents (García-Añoveros et al., 1997). ASIC channels are preferentially permeable to sodium (Na^+^), and to a lesser extent, other cations, such as potassium (K^+^), lithium (Li^+^), and proton (H^+^) (Fyfe et al., 1998). ASIC1a homotrimeric and ASIC1a/2b heterotrimeric channels are also permeable to calcium (Ca^2+^) (Yermolaieva et al., 2004; Sherwood et al., 2011). Thus, opening of these ASIC channels results in cation influx and neuronal activation. The different ASIC subunits have various acid activation threshold, leading to distinct pH sensitivity of ASIC channels based on their composition, which makes them more versatile in pH sensing even under conditions of dramatic pH changes.

The expression and distribution of different ASIC subunits remain unclear, because most currently available ASIC antibodies lack the needed specificity to differentiate them. Limited number of studies suggested that ASIC1a and ASIC2a/2b are the subunits mostly expressed in the central nervous system (Price et al., 1996; Waldmann et al., 1996; Lingueglia et al., 1997; Baron et al., 2008). In the peripheral nervous system, RNA for most ASIC subunits appears to be expressed in the human (Flegel et al., 2015) and rodent dorsal root ganglion (DRG) (Schuhmacher and Smith, 2016) with the exception of ASIC4 which has been either detected at very low level (Akopian et al., 2000) or not detected at all (Gründer et al., 2000). Similarly, electrophysiological experiments confirmed the presence of multiple types of ASIC currents in rodent DRG neurons (Mamet et al., 2002; Poirot et al., 2006). Using immunohistochemistry and *in situ* hybridization, the expression pattern of ASIC subunits, mainly ASIC1a and ASIC3, has been studied in rodent DRGs. ASIC1a and ASIC3 transcripts, as well as very low level of ASIC1b transcript were detected in TRPV1^+^ DRG neurons in rats (Ugawa et al., 2005). Another study also found that ASIC1a and ASIC3 appeared to be expressed in non-peptidergic neurons, which bind isolectin B4 (IB4) as well as in substance P-expressing afferents (Voilley et al., 2001). And while in mice, PCR experiments revealed that the most abundant ASIC subunit in DRG appeared to be ASIC3 (Wu et al., 2010; Schuhmacher and Smith, 2016), a detailed expression pattern of the different ASIC subunits remains largely unknown in this species.

The functional role of ASICs in nociception has been studied using pharmacological and genetic approaches. Intrathecal injection of PcTx1, a peptide from the venom of the Trinidad chevron tarantula, which specifically inhibits ASIC1a, was reported to reduce thermal, mechanical, and chemical nociception, as well as chronic inflammatory and neuropathic pain in rodents (Duan et al., 2007; Mazzuca et al., 2007). Another peptide, mambalgin-1, which was obtained from the black mamba venom, could inhibit currents of multiple types of ASIC channels with different composition (Diochot et al., 2012). Intrathecal or systemic intravenous injection of mambalgin-1 reduced both thermal and mechanical inflammatory pain (Diochot et al., 2016). Moreover, injecting mambalgin-1 into the paw also reduced acute thermal pain (Diochot et al., 2012). Thus, both central and peripheral ASIC subunits seem to play an essential role in physiological and pathological pain. However, behavioral studies using a knockout approach, where a specific ASIC subunit was deleted, reported either no detectable phenotype or conflicting results with respect to phenotype (Sluka et al., 2003; Drew et al., 2004; Roza et al., 2004; Staniland and McMahon, 2009; Walder et al., 2010), raising questions on the role of ASICs in somatosensation. Furthermore, the expression pattern of ASIC subunits in nerve injury models of neuropathic pain has not been investigated yet. Their role in pathological pain thus remain elusive.

In this study, we used an *in situ* hybridization approach (RNAscope) to overcome the obstacle of poor specificity of other detection methods. We performed quantitative assessment of the expression pattern of ASIC subunits in adult male mouse DRG. Our results reveal that the five ASIC subunits, including ASIC1a, ASIC1b, ASIC2a, ASIC2b, and ASIC3 have distinct distribution pattern among different populations of DRG neurons. In nerve injury condition, changes in ASIC subunits seem to occur in separate populations of afferents. Furthermore, the pattern of change varied among DRG depending on their segmental level, pointing to differential regulatory mechanisms between afferent types and anatomical location.

## Materials and Methods

### Animals

Adult male C57BL/6 wild-type mice between 20 and 25 g were purchased from Charles River Laboratories and The Jackson Laboratory and used for all the experiments. Three to four animals were housed per cage, maintained on a 12:12 h day: night cycle and had access to water and food *ad libitum*. All behavioral experiments were carried out during the light cycle. All experiments were performed in accordance with the regulations of the Canadian Council on Animal Care and approved by the Laval University Animal Care Committee.

### Peripheral Nerve Injury Model

Cuff surgery was used as a peripheral nerve injury model. It was performed by implanting a polyethylene cuff around the sciatic nerve as described previously (Mosconi and Kruger, 1996; Coull et al., 2003). Briefly, mice were anesthetized with isoflurane (between 1.9 and 2.2%). Under aseptic conditions, the sciatic nerve, of both sides, was exposed, one at a time, and carefully freed from surrounding tissue. A 2 mm section of split PE-50 polyethylene tubing was placed around the sciatic nerves (Cuff group). Then, the shaved skin was carefully stitched with surgical knots. For the sham-operated animals, the same procedure was applied without insertion of the polyethylene tubing (Sham group). The mechanical sensitivity of the animals was measured before and three to four weeks after surgery. A significant decrease of the paw withdrawal threshold after nerve injury was considered as indicating development of mechanical allodynia. All nerve-injured mice, but none of the sham mice, developed mechanical allodynia after the surgery.

### Mechanical Sensitivity

Mice were housed at the animal facility for at least 10 days prior to any behavioral testing. Each mouse was placed individually in a plexiglass chamber with a wire mesh floor and allowed to habituate for one hour before starting the test. Mechanical sensitivity threshold of the hind paws was determined with the SUDO method (Bonin et al., 2014) using calibrated von Frey monofilaments (0.04, 0.07, 0.16, 0.4, 0.6, 1.0, and 2.0 g). Both hind paws were tested and the threshold from both hind paws was averaged.

### *In situ* Hybridization (RNAscope) and Co-immunohistochemistry

Mice were deeply anesthetized by intraperitoneal injection of Urethane (30% in saline; 0.1 ml/10 g body weight) and then perfused transcardially with saline (0.9% NaCl), followed by 4% paraformaldehyde and 0.1% picric acid in PB (0.1M KH_2_PO_4_, 0.085M NaOH; pH 7.4). All the solutions were prepared with H_2_O pre-treated with DEPC (D5758, Sigma-Aldrich) to eliminate RNase.

Lumbar DRGs from naïve animals, and L4 and L5 DRGs from nerve-injured and sham animals were dissected and postfixed in the same fixation solution for two hours at 4°C. Afterwards DRGs were transferred to 30% sucrose solution at 4°C for at least 24 h for cryoprotection before they were sectioned into 10 μm thick slices using a cryostat (CryoStar NX50 Cryostat, Thermo Scientific). Sections were placed on Superfrost Plus glass slides (Fisher Scientific) and dried on a slide warmer (XH-2004, Premiere) at 40°C for 1 h. Then, they were stored at −30°C until further use. DRG sections from sham and nerve-injured animals were processed simultaneously on the same glass slide.

Prior to the employment of the RNAscope technique (Wang et al., 2012) from Advanced Cell Diagnostics (ACD) for *in situ* hybridization, we validated the procedure using positive and negative control probes provided by the manufacturer. The positive control probes include Polr2a, Ppib, and Ubc (320881, ACD), while the negative control probe is DapB of *Bacillus subtilis* strain (320871, ACD). All the probes were visualized with the RNAscope Fluorescent Multiplex Assay (Multiplex Assay; 320850, ACD) using naïve mouse DRG sections. For most experiments, we used the RNAscope 2.5 HD Assay - RED (Red Assay; 322360, ACD) which is the one that can be combined with immunohistochemistry as per the manufacturer’s guidelines. We also used the Multiplex Assay to study the co-expression of ASIC1a, ASIC2b and ASIC3.

The *in situ* hybridization with RNAscope Red Assay has been previously described (Wang et al., 2018). Briefly, glass slides with DRG sections were heated on the slide warmer at 60°C for 30 min and then were treated with Protease Plus (322330, ACD) for 20 min at 40°C. The sections were incubated with either ASIC1a (462381, ACD), ASIC1b (474591, ACD), ASIC2a (480571, ACD), ASIC2b (489031, ACD) or ASIC3 (480541, ACD) probes for two hours at 40°C. Afterward, the signal was revealed following the manufacturer’s instructions. The incubation time of Amplifier 5 was adjusted empirically to optimize the signal intensity for different probes: 40 min for ASIC1a, ASIC1b, ASIC2a, and ASIC2b; 30 min for ASIC3.

After *in situ* hybridization, the sections were washed with PBST (0.022M NaH_2_PO_4_, 0.08M Na_2_HPO_4_, 0.15M NaCl, and 0.15% Triton; pH 7.4). Then the sections were incubated with one of the primary antibodies against NF200 (1:500; ab4680, Abcam) or CGRP (1:1000; C8198, Millipore Sigma), or with IB4 conjugated Alexa Fluor 647 (1:200; I32450, Thermo Fisher Scientific) at 4°C overnight. The IB4 dye and CGRP primary antibodies were diluted in PBST containing 4% normal goat serum. The NF200 primary antibody was diluted at 1:500 in PBS (0.001M NaH_2_PO_4_, 0.0.3M Na_2_HPO_4_, 0.155M NaCl; pH 7.4) containing 0.3% Triton and 1% Bovine Serum Albumin (A3160501, Thermo Fisher Scientific). Then the sections were washed three times with PBST and incubated with secondary goat anti-chicken Alexa 647 (1:200; A21449, Invitrogen), goat anti-rabbit Alexa 647 (1:200; A21245, Invitrogen) or IB4 conjugated with Alexa 647 (1:200; I32450 Thermo Fisher Scientific) respectively for 2 h at 37°C. All the secondary antibodies and dye were diluted in PBST. For the double histochemistry, goat anti-rabbit Alexa 488 (1:200; A11008, Invitrogen) and IB4 conjugated with Alexa 647 (1:200; I32450 Thermo Fisher Scientific) were used. The sections were washed three times with PBST and mounted with Fluoroshield Mounting Medium containing DAPI (ab104139, Abcam).

The RNAscope Multiplex Assay was conducted following the company’s guidelines. Briefly, DRG sections were heated on the slide warmer at 60°C for 30 min and then treated with Protease III (322340, ACD) at 40°C for 20 min. Then a mixture of ASIC1a (462381, ACD), ASIC2b (489031-C2, ACD), and ASIC3 (480541-C3, ACD) probes was prepared (50:1:1 dilution, as per the company’s guidelines) and incubated with DRG sections for 2 h at 40°C. Afterwards the signal was revealed using the Multiplex Assay following the manufacturer’s instructions. Amplifiers 1, 2, 3, and 4 were incubated for 30, 15, 30, and 15 min respectively. We used the Amplifier 4 – alt A for all the experiments (Channel 1 = Alexa 488, Channel 2 = Atto 550, Channel 3 = Atto 647).

### Image Acquisition and Analysis

Images were captured using a confocal microscope (Zeiss, LSM 700; Oberkochen, Germany) equipped with a 20x, 40x, and 63x objective. The microscope settings (laser power, gain, and offset) were fixed in a given imaging session and kept unchanged for each gene of interest (ASICs). Images were, then, processed using the ZEISS ZEN software. The number of labeled neurons was counted using ImageJ (v 1.50b, NIH, United States) and a neuron was considered ASIC positive if the experimenter could observe three or more puncta- or cluster-like structures, which is consistent with the quantification guideline from ACD. To analyze the images from sections processed with the Multiplex Assay, we used a custom-built MATLAB (MathWorks) code to accelerate the process and blind the experimenter from knowing the gene under analysis. The experimenter was blind to the condition of animals during the analysis of the images from nerve-injured and sham animals.

To measure the neuronal cross-sectional area of ASIC/NF200-double positive neurons, polygon ROIs were drawn to outline ASIC/NF200-double positive neurons which also displayed clear nucleus based on the NF200 staining. The drawing and measuring the size of the polygon ROI were conducted using ImageJ. The same method was applied to measure the cross-sectional area of CGRP^+^ and ASICs/CGRP-double positive neurons in sham and nerve-injured conditions.

### Real-Time qPCR

Global ASICs gene expression levels were measured in the L4, L5, and L6 DRG of nerve-injured and sham animals. The DRGs from two or three animals were combined as one sample to get appropriate amount of RNA. Total RNA was extracted with Trizol reagent (15596026, Invitrogen) and reverse transcribed with Maxima H Minus First Strand cDNA Synthesis Kit (k1651, Thermofisher Scientific). Real-time qPCR was performed using a LightCycler 480 Instrument II (Roche) with LightCycler 480 SYBR Green I Master (04707516001, Roche). The sequences of primers were adapted from Schuhmacher and Smith (2016) and are listed in Table 1. The protocol consisted of a pre-incubation step at 95°C for 5 min and the amplification was realized for 45 cycles with denaturation at 95°C for 10 s, followed by annealing at 62°C for 30 s, and extension at 72°C for 10 s. All samples were measured in duplicates in a 96-well PCR Microplate. GAPDH was used as an internal control.

**TABLE 1:** Primer sequences used for real time qPCR experiment.

	Forward	Reverse
ASIC1a	GAACTGAAGACCGAGGAGGAG	GCCGCTCATAGGAGAAGATGT
ASIC1b	TCAGCTACCCTGACTTGCTCTA	GAGCGGTTGTAGAAACGATGGA
ASIC2a	CGATGGACCTCAAGGAGAGC	ATACACGAAGATGTGGCGGAT
ASIC2b	CTTGCTGTTGTCCTGGTCCT	TTGTTGTTGCACACGGTGAC
ASIC3	TTCACCTGTCTTGGCTCCTC	TGACTGGGGATGGGATTTCTAAG
GAPDH	TGTGAACGGATTTGGCCGTA	ACTGTGCCGTTGAATTTGCC

### Simulation of the Relationship Between the Number of Puncta Detected vs. the Puncta Density

We generated simulations to test the accuracy of detection of mRNA puncta in fluorescence images in comparison with measuring fluorescence intensity. First, circular regions of interests of 25 μm of diameter were generated to simulate average mouse DRG neurons. Depending on their density, various numbers of point-emitters as puncta were randomly distributed across an integer matrix covering the circular ROI. Multiple particles could occupy the same position or overlap with each other, and each particle contributes a fluorescent value of one. Parameter ε is defined as the mean intensity counts detected from a particle within the effective focal volume. Each value in the integer matrix was multiplied by the product of ε and the area of a disk of radius ω_*0*_, where ω_*0*_ is the *e^–2^* radius of the Gaussian convolution function simulating the point spread function (PSF) of the confocal microscope, $P\,S\,F\,\left(x,y\right)=I_{0}\cdot e^{-2\,\left(x^{2}+y^{2}\right)/\omega_{0}^{2}}$. The simulations assume linearity in the detected signal (*i.e.*, no saturation) and detection noise was added to all images to be more realistic. For the simulations, ε = 100 intensity units (i.u.), pixel size of 200 × 200 nm^2^, and ω_*0*_ = 300 nm were chosen. The puncta density was varied from 0.0001 to 3.1 puncta per μm^2^. The puncta were detected using a MATLAB (MathWorks) algorithm (*pkfnd*) that searches for maxima that have an intensity amplitude larger than a chosen threshold (6 i.u). By design, the distance between two detected peaks cannot be smaller than ω_*0*_. An analysis of the number of detected puncta/clusters and total intensity was then achieved.

### Intensity Analysis of ASIC Subunits mRNA Expression Across Neuronal Populations

The expression levels of ASIC subunits in individual neurons were studied by measuring the mean intensity of ASIC signal within CGRP^+^ or IB4^+^ neurons. To do so, the experimenter placed a point-shape ROI close to the center of ASICs/IB4- or ASICs/CGRP-double positive neurons. Then a circular binary region (10 pixels/6.3 μm in radius) centered on each ROI was created automatically inside individual neuron. The mean intensity of ASIC signal within the region was calculated to represent the expression level of ASIC subunit in that neuron. Meanwhile, three to five regions within the tissue without presence of detectable puncta were manually selected, and their mean intensity were averaged as mean background for this image. This value was then subtracted from the signal measured in each neuron from the same image.

### Statistical Analysis

All results are presented as means ± standard error of the mean (SEM) unless stated otherwise. GraphPad Prism 7 (GraphPad Software) was used for all statistical analysis and the criterion for significance was set at *p* < 0.05.

## Results

### Expression Pattern in Adult Naïve Male Mice

The distribution of different ASIC subunits was examined in sections of lumbar mouse DRG using the RNAscope Red Assay for *in situ* hybridization. The sensitivity and selectivity of the technique were tested with positive and negative control probes (Supplementary Figure S1). Reference probes used for comparison included: Ubiquitin C (UBC), Peptidylpropyl isomerase B (PPIB), DNA-directed RNA polymerase II subunit RPB1 (POLR2A), and bacillus subtilis dihydrodipicolinate reductase (DapB). These were chosen because they represent canonical markers with high, medium, low, and no expression levels, respectively, based on patterns observe in a wide variety of tissues (Wang et al., 2012; Li and Kim, 2015). The signal levels of these positive (Supplementary Figure S1A) and negative (Supplementary Figure S1B) control probes in mouse DRG sections was consistent with that reported in other tissues, providing validation of the sensitivity of the technique in our experimental conditions.

To determine the distribution of ASIC subunits among different subpopulations of DRG neurons, we performed immunohistochemistry following *in situ* hybridization. We targeted three distinct populations based on the following markers: neurofilament 200 (NF200), isolectin B4 (IB4), and calcitonin gene-related peptide (CGRP). NF200 was used as a marker of myelinated afferents associated with medium- to large-diameter DRG neurons. These neurons are believed to be involved in nociception, tactile perception, and proprioception (Kuniyoshi et al., 2007; Ruscheweyh et al., 2007; Luo et al., 2009). CGRP was used as a marker of unmyelinated peptidergic afferents, while IB4 was used to label unmyelinated non-peptidergic afferents. Both populations of unmyelinated afferents are associated with small-diameter DRG neurons and mainly correspond to nociceptors (Averill et al., 1995; Molliver et al., 1995; Snider and McMahon, 1998).

**FIGURE 1:**
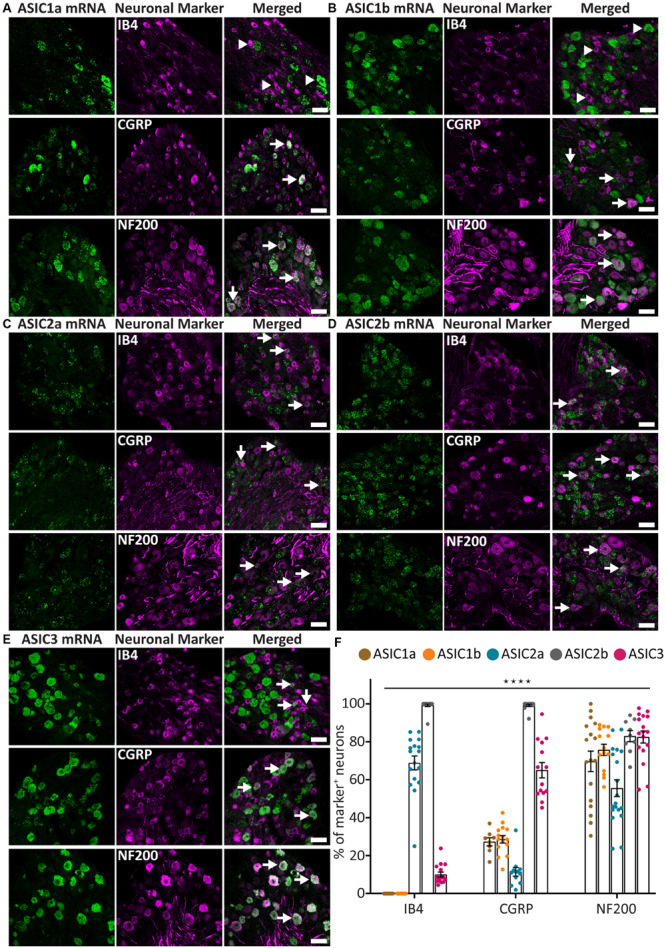
ASICs expression in naïve adult mouse DRG. **(A–E)** Representative confocal images showing the presence of ASIC1a **(A)**, ASIC1b **(B)**, ASIC2a **(C)**, ASIC2b **(D)**, and ASIC3 **(E)** transcripts in DRG neurons with different neuronal markers, including IB4, CGRP, and NF200. **(F)** Percentage of the marker positive neurons expressing different ASIC subunits. Arrow heads point to neurons expressing only ASIC1a, ASIC1b, or IB4. Arrows point to ASIC/marker-double positive neurons. The expression pattern of the five ASICs subunits was significantly different among the three neuronal subpopulations (****p < 0.0001, using two-way ANOVA). Scale bar = 50 μm; *n* = 9–19 DRGs from 3 to 4 naïve mice.

The probes for five ASIC subunits, including ASIC1a, ASIC1b, ASIC2a, ASIC2b, and ASIC3, all gave moderate to strong signal with distinct distribution (Figure 1). We observed both puncta and clusters, the latter being the fusion of multiple puncta in high density. Among the ASICs, ASIC1a and ASIC1b displayed similar pattern, as they were present in around 28% of CGRP^+^ neurons and around 70% of NF200^+^ neurons (Figures 1A,B,F). However, they were not detected in any IB4^+^ neurons (Figures 1A,B,F).

On the contrary, ASIC2a and ASIC2b displayed very different expression patterns. ASIC2a was expressed in around 70% of IB4^+^ neurons and in about half of the NF200^+^ neuron, but only present in about 10% of CGRP^+^ neurons (Figures 1C,F). On the other hand, ASIC2b was detected in almost all DRG neurons, including 100% of CGRP^+^ and IB4^+^ neurons, and more than 80% of NF200^+^ neurons (Figures 1D,F).

The ASIC3 probe displayed the strongest signal compared to other subunits (Figure 1E), which is in accordance with previous studies (Hughes et al., 2007; Deval et al., 2011; Schuhmacher and Smith, 2016). ASIC3 was expressed in most NF200^+^ neurons (73%) and CGRP^+^ neurons (65%), but only in a few IB4^+^ neurons (10%; Figures 1E,F).

Overall, the five ASIC subunits, except for ASIC1a and ASIC1b, showed a distinct expression pattern within the three populations of DRG neurons studied, indicating that different ASIC subunits might be involved in different somatosensation.

### Size Distribution of ASIC/NF200-Double Positive Neurons

Given that all five ASIC subunits are expressed in a majority of NF200^+^ neurons, and that NF200^+^ neurons represent morphologically and functionally heterogeneous populations, we further investigated whether ASIC subunits are present in different subpopulations of NF200^+^ neurons by characterizing the soma size of ASIC/NF200-double positive neurons.

**FIGURE 2:**
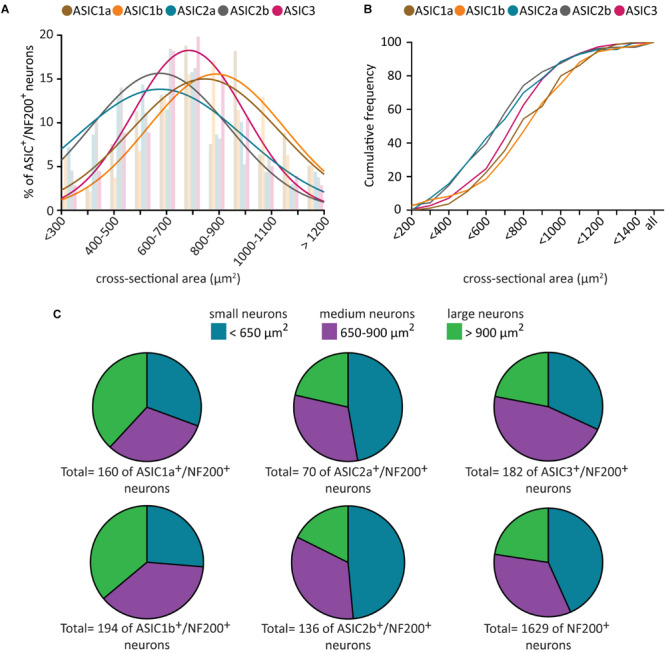
Size distribution analysis of ASICs/NF200-double positive neurons. Histogram **(A)** and cumulative curves **(B)** of the neuronal size distribution (bin = 100 μm^2^) of ASICs/NF200-double positive neurons in naïve adult mouse DRGs. Comparing to ASIC3/NF200-double positive neurons, the distribution curves of ASIC2a/NF200- and ASIC2b/NF200-double positive neurons were significantly shifted to the left (*p* < 0.01 for ASIC2a/NF200 and *p* < 0.01 for ASIC2b/NF200 using Extra sum-of-squares *F*-test). In contrast, the distribution curve of ASIC1b/NF200-, but not ASIC1a/NF200-double positive neurons, was significantly shifted to the right (*p* < 0.01 for ASIC1b/NF200 and *p* = 0.20 for ASIC1a/NF200 using Extra sum-of-squares *F* test). **(C)** Pie charts showing the distribution of ASICs/NF200-double positive neurons in three different groups: small-diameter (<650 μm^2^), medium-diameter (650–900 μm^2^), and large-diameter neurons (>900 μm^2^). 70–194 neurons from 4 to 11 DRGs from 4 mice.

In naïve condition, the neuronal size of all five populations of ASIC/NF200-double positive neurons displayed a normal distribution (Figures 2A,B). The mean somal area for ASIC3/NF200-double positive neurons was between 700 and 800 μm^2^ (Figure 2A). The distribution of ASIC2a/NF200- and ASIC2b/NF200-double positive neurons showed a significant left shift in mean somal area, between 600 and 700 μm^2^. In contrast, only the distribution for ASIC1b/NF200- but not ASIC1a/NF200-double positive neurons, was significantly shifted to the right with a mean somal area between 800 and 1000 μm^2^ (Figure 2A). Both the distribution (Figure 2A) and cumulative probability plots (Figure 2B) showed that ASIC3/NF200-double positive neurons were smaller than ASIC1a/NF200- and ASIC1b/NF200-double positive neurons, but larger than ASIC2a/NF200- and ASIC2b/NF200-double positive neurons.

We further categorized these ASIC/NF200-double positive neurons into three groups based on their neuronal size. The neurons which were smaller than 650 μm^2^ were categorized as small-diameter neurons. Neurons with size between 650 and 900 μm^2^ were categorized as medium-diameter neurons, and neurons larger than 900 μm^2^ were categorized as large-diameter neurons (Wang et al., 2018). We found that around half of ASIC3/NF200-double positive neurons were medium-diameter neurons, and half of ASIC2a/NF200- and ASIC2b/NF200-double positive neurons were small-diameter neurons, while ASIC1a/NF200- and ASIC1b/NF200-double positive neurons were almost equally distributed among the three groups (Figure 2C). These data highlight a differential distribution pattern of ASIC transcripts among different subpopulation of NF200^+^ sensory neurons, further supporting the hypothesis that they might be involved in different types of somatosensation.

### ASIC1a, ASIC2b, and ASIC3 Co-expression in DRG Neurons

**FIGURE 3:**
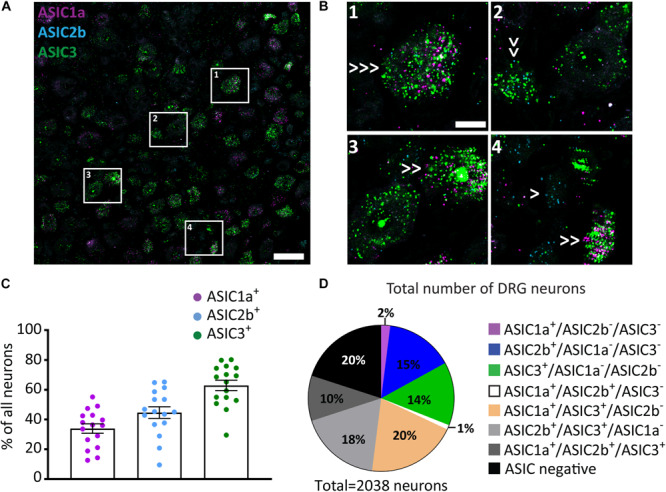
ASIC1a, ASIC2b, and ASIC3 co-expression in DRG neurons. **(A)** A representative confocal image from a DRG section showing expression of ASIC1a, ASIC2b, and ASIC3. Scale bar = 50 μm. **(B)** Images of the regions in left panel highlighted with white rectangles at high magnification. Single arrows point to cells expressing only one ASIC subunit, double arrows point to cells expressing two subunits, and triple arrows point to cells expressing all three subunits. Scale bar = 20 μm. **(C)** Percentage of all DRG neurons expressing different ASIC subunits. **(D)** Pie chart showing the distribution of DRG neurons expressing different combinations of ASIC1a, ASIC2b, and ASIC3; *n* = 16 DRG sections from 4 mice.

Our data showed distinct expression pattern of ASIC subunits across different populations of sensory neurons. We, then, asked what is the expression pattern of ASIC subunits in individual DRG neurons. To achieve this, we conducted *in situ* hybridization of multiple ASIC subunits using the RNAscope Fluorescent Multiplex Assay, which can detect up to three mRNAs simultaneously. ASIC1a, ASIC2b, and ASIC3 (Figures 3A,B) were chosen because ASIC1a and ASIC1b had very similar expression patterns, the expression pattern of ASIC2b subunit has not been explored in previous studies, and ASIC3 showed the strongest expression level in primary sensory neurons.

The Multiplex Assay revealed that around 35%, 50%, and 60% of all DRG neurons expressed ASIC1a, ASIC2b, and ASIC3, respectively (Figure 3C). When comparing results between the Red and the Multiplex Assays, we found that the percentage of ASIC1a^+^ and ASIC3^+^ neurons were similar; however, less ASIC2b^+^ neurons were observed with the latter assay. The discrepancy may be because the sensitivity of Multiplex Assay is lower than the Red Assay, thus, some ASIC2b weak positive neurons may have been missed by the Multiplex Assay.

Among all the 2038 counted neurons 15% and 14% of them expressed only the ASIC2b or only the ASIC3 subunit, respectively, while almost no DRG neurons (less than 2%) expressed the ASIC1a subunit alone (Figure 3D). Interestingly, the ASIC3 subunit was present in virtually all the DRG neurons that expressed multiple subunits: i.e., 20%, 18%, and 10% of all neurons were ASIC1a/ASIC3-double positive, ASIC2b/ASIC3-double positive, and ASIC1a/ASIC2b/ASIC3-triple positive, respectively. Meanwhile, less than 1% of all DRG neurons were ASIC1a/ASIC2b-double positive.

Although not all five ASIC subunits can be studied at once, due to the limitation of the approach, our *in situ* hybridization experiment using the Multiplex Assay already revealed complex combinations of ASICs subunits expressed inside individual neurons, indicating that heterotrimeric ASIC channels is the dominant form present in the majority of primary sensory neurons, and that ASIC3 is the most frequent subunit used to form these heterotrimeric channels.

Moreover, our *in situ* experiment with the Red Assay revealed that IB4^+^ neurons mainly expressed ASIC2a and ASIC2b, and to a lesser extent ASIC3. While CGRP^+^ neurons expressed all subunits to some extent, the main ones were ASIC2b and ASIC3. All five ASIC subunits were expressed in at least 50% of NF200^+^ neurons (Figure 1F). The results from the two different assays we used indicate that different types of sensory neurons express various combinations of ASICs subunits, yielding distinct sensitivity and responses to extracellular acidosis.

### Expression Pattern Analysis After Nerve Injury

**FIGURE 4:**
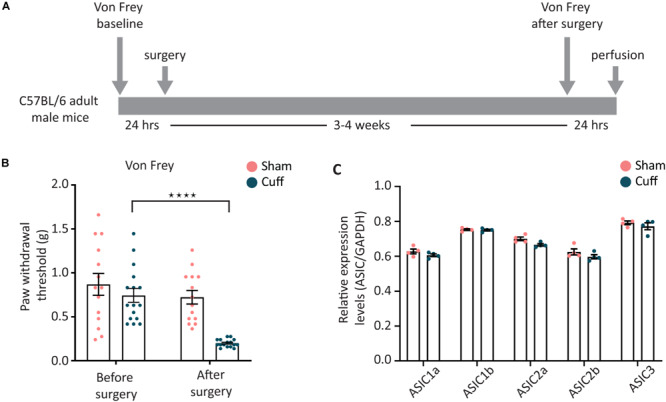
Overall expression levels of the different ASIC subunits were not altered after nerve injury. **(A)** Sciatic nerve cuff model was chosen to induce peripheral nerve injury. Mechanical sensitivity of plantar area of hind paws was measured before and after the surgery. **(B)** The paw withdrawal threshold of nerve-injured animals, but not sham animals, was significantly decreased after the surgery (*N* = 14 and 16 animals for the sham and cuff group, respectively; ****p < 0.0001 using two-way ANOVA test, followed by Sidak’s multiple comparisons test). **(C)** Real time qPCR results comparing the global mRNA levels of ASIC subunits between nerve-injured and sham animals; *N* = 4 for both sham and cuff groups. Each point represents a merger of L4, L5, and L6 DRGs of 2–3 animals of the same condition. No significant difference was found with two-way ANOVA test.

We, then, studied the regulation of ASIC subunits expression after nerve injury. The well-established sciatic nerve cuff model (Mosconi and Kruger, 1996; Coull et al., 2003) was used to induce peripheral nerve injury (Figure 4A). We confirmed the presence of mechanical allodynia before pursuing further experiments (Figure 4B).

Real-time PCR results showed that the overall expression level of all five ASIC subunits examined were not different between nerve-injured and sham animals in lumbar L4, L5, and L6 DRG segments when merged together (Figure 4C).

Nerve injury has been reported to have differential effects on separate DRG segments (Lindborg et al., 2018). Thus, we continued to analyze the expression of ASIC subunits in L4 and L5 DRGs separately, using the same approach as above – combining RNAscope *in situ* hybridization with histochemistry. We focused on IB4^+^ and CGRP^+^ neurons since they are the main subsets of nociceptors.

**FIGURE 5:**
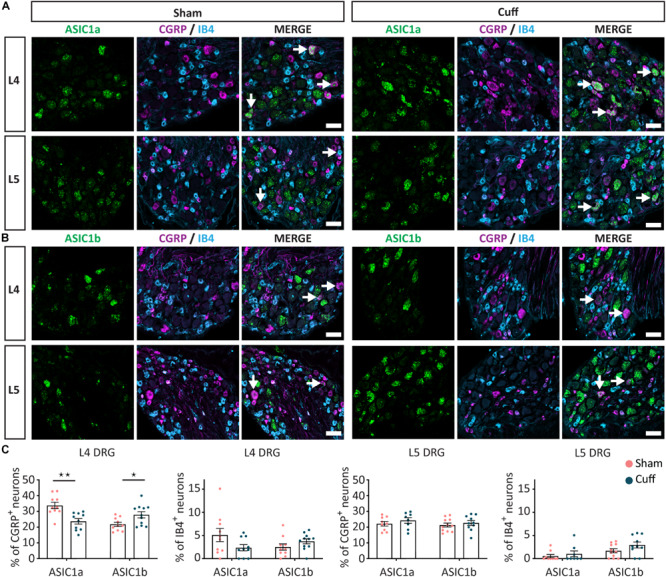
Change in ASIC1 expression in peptidergic neurons after nerve injury. **(A)** Representative confocal images showing ASIC1a **(A)** and ASIC1b **(B)**, with neuronal marker IB4 and CGRP in sham and nerve-injured animals in L4 and L5 DRGs. Arrows point to ASIC/marker-double positive neurons. **(C)** Nerve injury induced a down-regulation of ASIC1a, while an up-regulation of ASIC1b in peptidergic neurons only in L4, but not L5 DRGs. No significant difference was found in non-peptidergic neurons. *p < 0.05 and **p < 0.01 using unpaired *t*-test. Scale bar = 50 μm; *n* = 8–11 DRGs from 4 to 6 mice.

**FIGURE 6:**
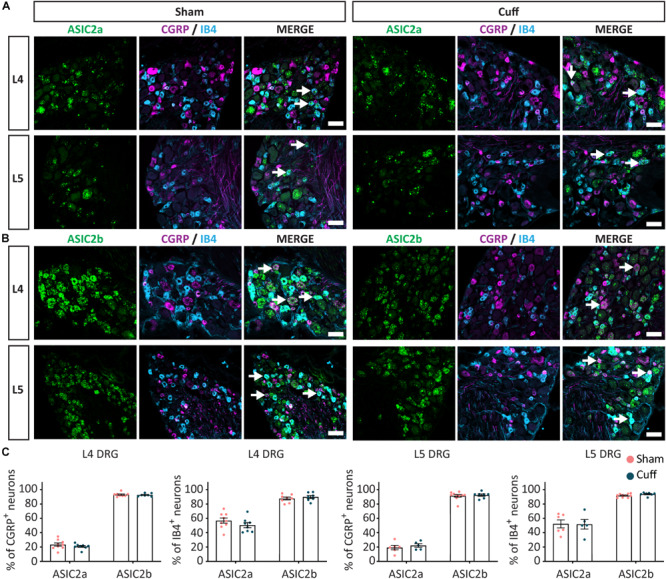
The expression pattern of ASIC2a and ASIC2b was unchanged after nerve injury. Representative confocal images showing ASIC2a **(A)** and ASIC2b **(B)**, with neuronal markers, IB4 and CGRP, in sham and nerve-injured animals in L4 and L5 DRG. Arrows point to ASIC/marker-double positive neurons. **(C)** The percentage of peptidergic and non-peptidergic neurons expressing ASIC2a or ASIC2b was unchanged after nerve injury, neither in L4, nor in L5 DRGs. Scale bar = 50 μm; *n* = 5–10 DRGs from 4 to 6 mice.

We found that in L4, but not L5 DRGs, the percentage of CGRP^+^ neurons expressing ASIC1a was significantly reduced in nerve-injured compared to sham animals (Figures 5A,C). In contrast, the percentage of CGRP^+^ neurons expressing ASIC1b was significantly increased in L4 but not L5 DRGs (Figures 5B,C). We did not observe any significant change in ASIC1a or ASIC1b in IB4^+^ neurons, neither in the L4 DRG, nor in the L5 DRG (Figures 5A–C). These data revealed opposite regulation of ASIC1a and ASIC1b, specifically in L4 peptidergic neurons. In contrast, ASIC2a and ASIC2b did not show any significant change in any of the population of neurons examined across L4 and L5 DRGs (Figures 6A–C).

**FIGURE 7:**
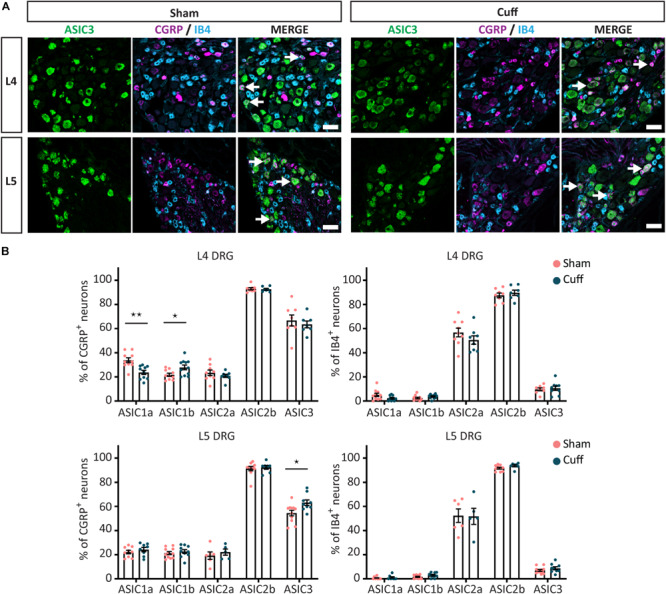
Quantitative analysis of the expression of ASIC subunits after nerve injury. **(A)** Representative confocal images showing ASIC3, with neuronal markers, IB4 and CGRP, in sham and nerve-injured animals in the L4 and L5 DRGs. Arrows point to neurons expressing ASIC3 and a neuronal marker (IB4 or CGRP). **(B)** Summary of the proportion of marker positive neurons expressing one of the ASIC subunits in sham and cuff mice. Part of the graph is a duplication of Figures 5C, 6C. *p < 0.05 and **p < 0.01 using unpaired *t*-test. Scale bar = 50 μm; *n* = 5–11 DRGs from 4 to 6 mice.

Finally, we found that the percentage of CGRP^+^ neurons expressing ASIC3 was significantly increased in L5 but not L4 DRGs (Figures 7A,B). In contrast, the percentage of non-peptidergic IB4^+^ neurons expressing ASIC3 was unaltered in either L4 or L5 DRGs (Figures 7A,B).

The fact that changes in ASIC1a, ASIC1b, and ASIC3 only occurred in CGRP^+^ neurons might be due to a change in cell types expressing CGRP after nerve injury. To test this possibility, we measured the neuronal size of CGRP^+^ sensory neurons and found no significant difference between nerve-injured and sham animals (Supplementary Figure S2A). Furthermore, the size of double positive (ASIC1a/CGRP in L4 DRG and ASIC3/CGRP in L5 DRG) neurons did not differ between nerve-injured and sham animals (Supplementary Figure S2B). Thus, the expression pattern of CGRP did not appear to alter after nerve injury.

Overall, our results showed that nerve injury induced differential regulation of ASICs expression in specific subpopulations of sensory neurons, which, in turn, varied among DRGs, indicating a complex regulatory mechanism for ASIC subunits expression in neuropathic pain condition.

### Expression Level Analysis Within Individual Cells After Nerve Injury

We continued investigating whether the amount of ASIC mRNA in individual CGRP^+^ and IB4^+^ neurons changed in our experimental conditions. Theoretically, the amount of mRNA can be quantified by either counting the number of puncta/clusters (Wang et al., 2012) or measuring the mean intensity of the fluorescent signal.

As shown before, all ASIC subunits displayed strong signal with high density of puncta/clusters (Supplementary Figure S3A). In principle, when the density of puncta increases, the number of distinguishable puncta will be severely underestimated. This effect can be observed in our simulated data in which the number of detected puncta saturated and deviated from the number of existing puncta as the density increased (Supplementary Figures S3B,C). In contrast, the total intensity in the image scales linearly with the number of existing puncta (Supplementary Figure S3D). This is guaranteed since the detection saturation in our experiments is minimized by carefully choosing appropriate imaging parameters (e.g., PMT gain, laser intensity, and pixel dwell time). Thus, counting number of puncta/clusters will lead to large amount of error. We therefore opted to measure the mean intensity of ASIC subunit signal to represent the level of mRNA expression.

Prior to employing intensity measurement, we compared three segmentation approaches based on CGRP labeling: in the first one, CGRP^+^ neurons (Supplementary Figure S4A1) were manually selected by the experimenter and circular ROIs were placed close to the center of these CGRP^+^ neurons (Supplementary Figure S4A2). The second approach was a semi-automated approach where the dynamic ROIs covering the whole neuron were automatically drawn on CGRP^+^ neurons selected by the experimenter (Supplementary Figure S4A3). The third approach used a random forest algorithm to fully automatically segment CGRP^+^ neurons with dynamic ROIs covering the whole neuron (Supplementary Figure S4A4). The mean intensity of ASIC signal inside the ROIs generated from these three approaches was measured and statistical analysis showed that they yielded similar results (Supplementary Figure S4B).

However, the fully automated approach falsely identified some round blood vessels as neurons which resulted in higher false positive rate (more ROIs were generated than manual selection) and it also demonstrated difficulty in separating two touching neurons. Furthermore, it was extremely time consuming. The semi-automated approach would yield the mean intensity from the whole neuron, but it required extensive manual inspection to ensure that the drawing was adequate, and it also became very time consuming. Importantly, there was a high linear correlation between the mean intensity calculated from circular and dynamic ROIs from the positive neurons selected by the experimenter (Supplementary Figure S4C). Thus, the first approach can be used to replace the semi-automated approach with time advantage and was chosen for our analysis.

**FIGURE 8:**
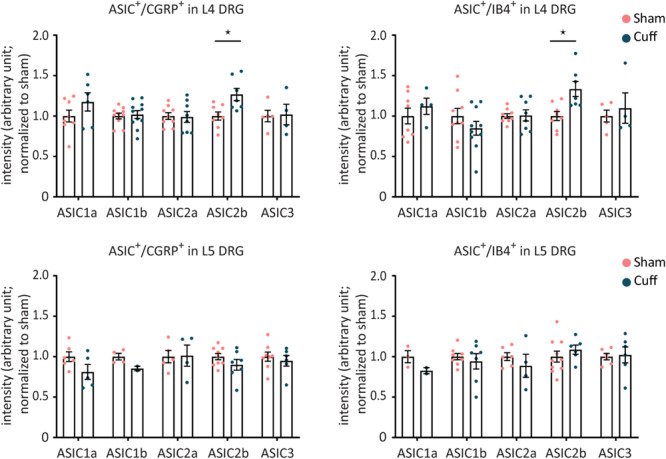
The mRNA expression levels in individual CGRP^+^ or IB4^+^ neurons were not altered for any of the ASIC subunits, except for ASIC2b, after nerve injury. Intensity analysis results of ASIC1a, ASIC1b, ASIC2a, ASIC2b, and ASIC3 in individual CGRP^+^ or IB4^+^ neurons in L4 and L5 DRGs. *p < 0.05 using one-way ANOVA test, followed by Tukey’s multiple comparisons test; *n* = 2–11 DRGs from 4 to 6 mice.

To investigate whether the amount of ASIC mRNA changed in CGRP^+^ and IB4^+^ neurons after nerve injury, the experimenter manually selected ASIC/CGRP- or ASIC/IB4-double positive neurons and measured mean intensity of ASIC signal. The analysis results did not reveal any significant difference between the two groups (nerve-injured and sham animals) except for ASIC2b (Figure 8). The mRNA expression levels of ASIC2b were significantly increased in both CGRP^+^ and IB4^+^ neurons within the L4, but not L5 DRG, of the nerve-injured group compared to sham group (Figure 8).

## Discussion

In the present study we demonstrated that ASIC subunits expressed in adult mouse DRG show a differential expression pattern among distinct neuronal populations. Considering the fundamental role ASIC subunits play in nociception, from peripheral transduction (Yu et al., 2010; Bohlen et al., 2011) to the development of central sensitization and pain hypersensitivity (Holzer, 2009) we proceeded to study how peripheral nerve injury affects the expression pattern of different ASIC subunits. We found a regional and cell type specific differential regulation of ASIC subunits.

Among the different ASIC subunits, ASIC1a and ASIC1b showed very similar mRNA expression pattern. Consistent with this observation, a recent study, also using RNAscope, showed a high level of ASIC1a/1b co-expression in unidentified DRG neuronal populations in mice (Chang et al., 2019). Together, these findings suggest that the efficiency of alternative splicing for ASIC1a and ASIC1b is comparable in normal conditions. Yet, we found that, after nerve-injury, the regulation of ASIC1a and ASIC1b expression went in opposite directions within the CGRP population, suggesting a shift in splicing toward ASIC1b in this cell type. Although changes in alternative splicing efficiency have been reported in various disease models (Wang and Cooper, 2007), to our knowledge, our study is the first to report potential regulation of alternative splicing after nerve-injury. Alteration in splicing may be an important molecular mechanism underlying neuropathic pain and should therefore be further explored.

In this study, we found that ASIC3 is expressed in a majority of NF200^+^ neurons (73%) and CGRP^+^ neurons (65%), but only in few IB4^+^ neurons (10%). It has been reported, using an Asic3-knockout/eGFP-f-knockin mouse line, that ASIC3 expressing neurons accounts for ∼30% of all lumbar DRG neurons, including 27% of CGRP^+^ and 23% of IB4^+^ neurons, as well as 50% of N52^+^ myelinated neurons (Lin et al., 2016). We found a higher rate of ASIC3/CGRP-double positive neurons using *in situ* hybridization, possibly reflecting higher sensitivity of mRNA detection using the RNAscope Red assay.

The expression of ASIC4 in DRG neurons has been reported either at very low level (Akopian et al., 2000) or not detected at all (Gründer et al., 2000). Furthermore, ASIC4 has been hypothesized to only play a regulatory role, given that it does not form functional homomeric channels (Akopian et al., 2000), thus we decided not to include this subunit in our study.

We also investigated the co-expression of multiple ASIC subunits within individual DRG neurons, which had not been explored before. Our observations demonstrated that the majority of possible combinations of ASIC1a, ASIC2b, and ASIC3 are present in lumbar DRG neurons, except that very few ASIC1a-single positive and ASIC1a/ASIC2b-double positive neurons were found. Our results were consistent with previous electrophysiological data showing that acid-sensitive currents recorded from thoracic and cardiac sensory neurons were mainly mediated by heterotrimeric ASIC channels (Sutherland et al., 2001; Hughes et al., 2007). The overall co-expression pattern of the five ASIC subunits is probably even more complicated than what our results revealed, but this could not be explored further with our approach since only three ASIC subunits can be detected simultaneously with the Multiplex Assay.

Several previous studies have investigated the overall expression levels of ASIC subunits in various models of pain hypersensitivity and species. While these studies did not focus on specific cell populations, it remains interesting to contrast their findings with ours, as far as global levels of subunit expression are concerned. For example, our real time PCR analysis revealed no significant overall change for the five ASIC subunits in the cuff model of nerve injury in mice. In contrast, two other nerve injury models in rats, spinal nerve injury (SNI) and spinal nerve ligation (SNL), yielded differential regulation of ASIC transcripts: down-regulation of ASIC1a and ASIC1b in SNI vs. up-regulation of all ASIC subunits, except ASIC2b, in SNL (Poirot et al., 2006). In addition, previous studies reported up-regulation of ASIC3 protein in rat DRGs in inflammation models (Ohtori et al., 2006). Finally, another study reported significant increase of ASIC2 and ASIC3, but not ASIC1 mRNA in a mouse model of inflammation (Walder et al., 2010). The differences observed in the regulation of the overall expression of ASICs in various pain models suggests that ASICs may play distinct roles in chronic pain depending on the condition.

Beyond examining global mRNA levels, our study highlights differential regulation of ASIC subunits among different subpopulation of afferents as well as DRG segments after nerve injury. For example, a rise in ASIC1b in L4 DRG and ASIC3 in L5 DRG vs. a decrease in ASIC1a in L4 DRG; all these occurred specifically in CGRP^+^ neurons. When considering intensity per cell, ASIC2b showed an increase in both CGRP^+^ and IB4^+^ neurons, specifically in L4 DRG. Such DRG-specific change has been reported for other markers (Laedermann et al., 2014). These findings highlight the importance of conducting quantification in specific neuronal populations and DRG segments because opposite regional or cell-specific changes can be missed when conducting global tissue measurements.

Different ASIC channels have distinct pH sensitivity and unique kinetic properties upon activation depending on the combination of ASIC subunits that form a functional channel (Benson et al., 2002; Hesselager et al., 2004). For instance, it has been documented that homotrimeric ASIC1a, ASIC1b, and ASIC2a channels mediate a transient current with rapid adaptation (Gründer and Pusch, 2015) whereas ASIC3 channels show biphasic kinetics including a transient component followed by a sustained component (Delaunay et al., 2012). This sustained current is associated with a very slow inactivation kinetics (Salinas et al., 2009). This slow component has been suggested to play an important role in hypersensitivity following peripheral inflammation (Deval et al., 2003, 2008; Yagi et al., 2006). Hence, after peripheral nerve injury, the upregulation of ASIC3 in CGRP^+^ L5 DRG neurons may enable ASIC channels to stay active even in acidic conditions caused by the injury. This may yield prolonged acid-induced current and hypersensitivity. However, since our data (Figure 3) and previous studies (Benson et al., 2002; Hattori et al., 2009) show that heterotrimeric channels are the dominant form of native ASIC channels, it is difficult to precisely predict the stoichiometry of the channels that result from changes in expression after nerve injury. Nevertheless, it is likely that the changes in expression patterns we observed, can lead to changes in channel composition and functional properties (Poirot et al., 2006). It would be intriguing to test our hypotheses by using electrophysiology to record ASIC currents in DRG neurons from nerve-injured mice, and draw links between changes in expression pattern and function.

## Data Availability Statement

The datasets generated for this study are available on request to the corresponding author.

## Ethics Statement

The animal study was reviewed and approved by Canadian Council on Animal Care and Laval University Animal Care Committee.

## Author Contributions

MP-T, FW, and YD designed the study, analyzed the data, and wrote the manuscript. MP-T performed all the experiments. SL and AG developed analytical tools to assist the intensity analysis of the RNAscope images and validate the methodological approach. FW and YD supervised the research.

## Conflict of Interest

The authors declare that the research was conducted in the absence of any commercial or financial relationships that could be construed as a potential conflict of interest.
