# Supplementary material for: Differential Expression of Acid – Sensing Ion Channels in Mouse Primary Afferents in Naïve and Injured Conditions
Source: Front Cell Neurosci. 2020 May 19;14:103. doi: 10.3389/fncel.2020.00103 (PMC7248332; doi:10.3389/fncel.2020.00103)
Supplement: Supplementary file 1 [file Image_1.pdf]

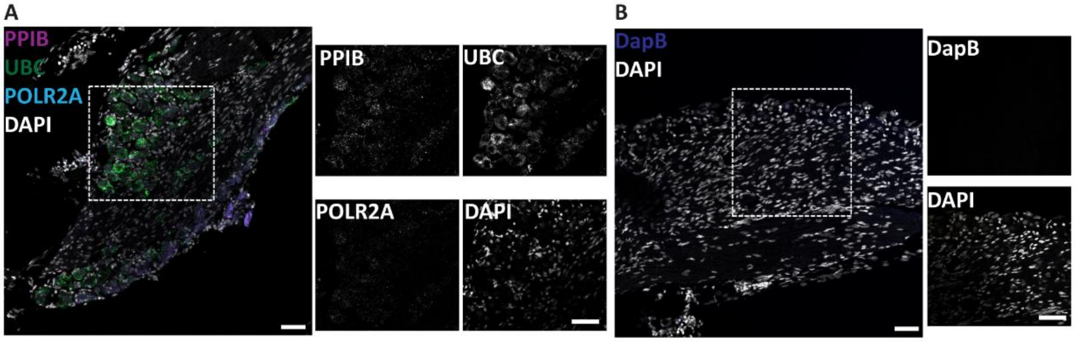

SUPPLEMENTARY FIGURE 1: Validation of the RNAscope approach. **(A)** The signal levels of positive control probes targeting eukaryotic housekeeping genes Polr2a, Ppib, and Ubc, respectively, were consistent with our expectations. **(B)** No detectable signal was found when the negative control probe targeting bacterial gene DapB was used. In both (A) and (B), the left panels are showing representative confocal images acquired with 20x objective. The images in right panels were acquired with 40x objective from the area highlighted in the left panel to have higher magnification. For both experiments, DRG sections from naïve adult male mice were used. Scale bar = 50 μm.

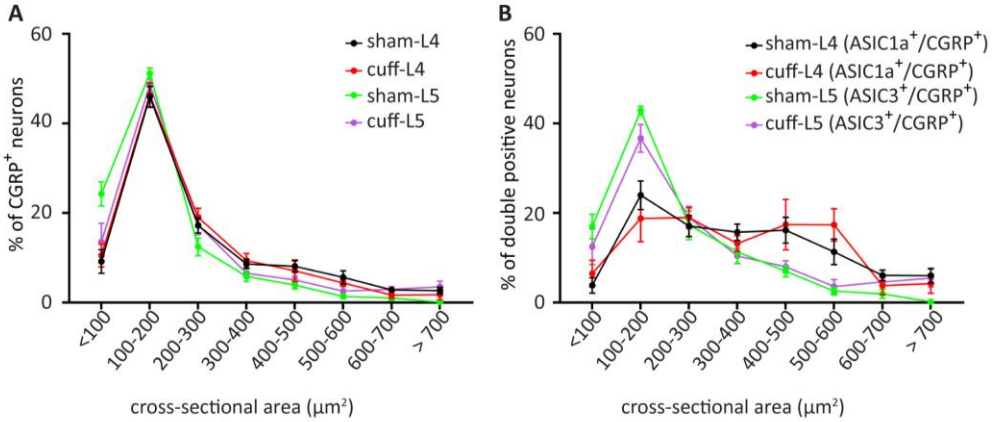

SUPPLEMENTARY FIGURE 2: The size distribution of $CGRP^+$ and ASICs /CGRP-double positive neurons was not different between nerve-injured and sham animals. Neuronal size distribution of $CGRP^+$ neurons **(A)**, and ASIC1a/CGRP- and ASIC3/CGRP-double positive neurons **(B)** in L4 and L5 DRGs of nerve-injured and sham animals (bin = 100 μm^2). No significant difference was found using two-way ANOVA test. n = 4-8 DRGs from 4 mice.

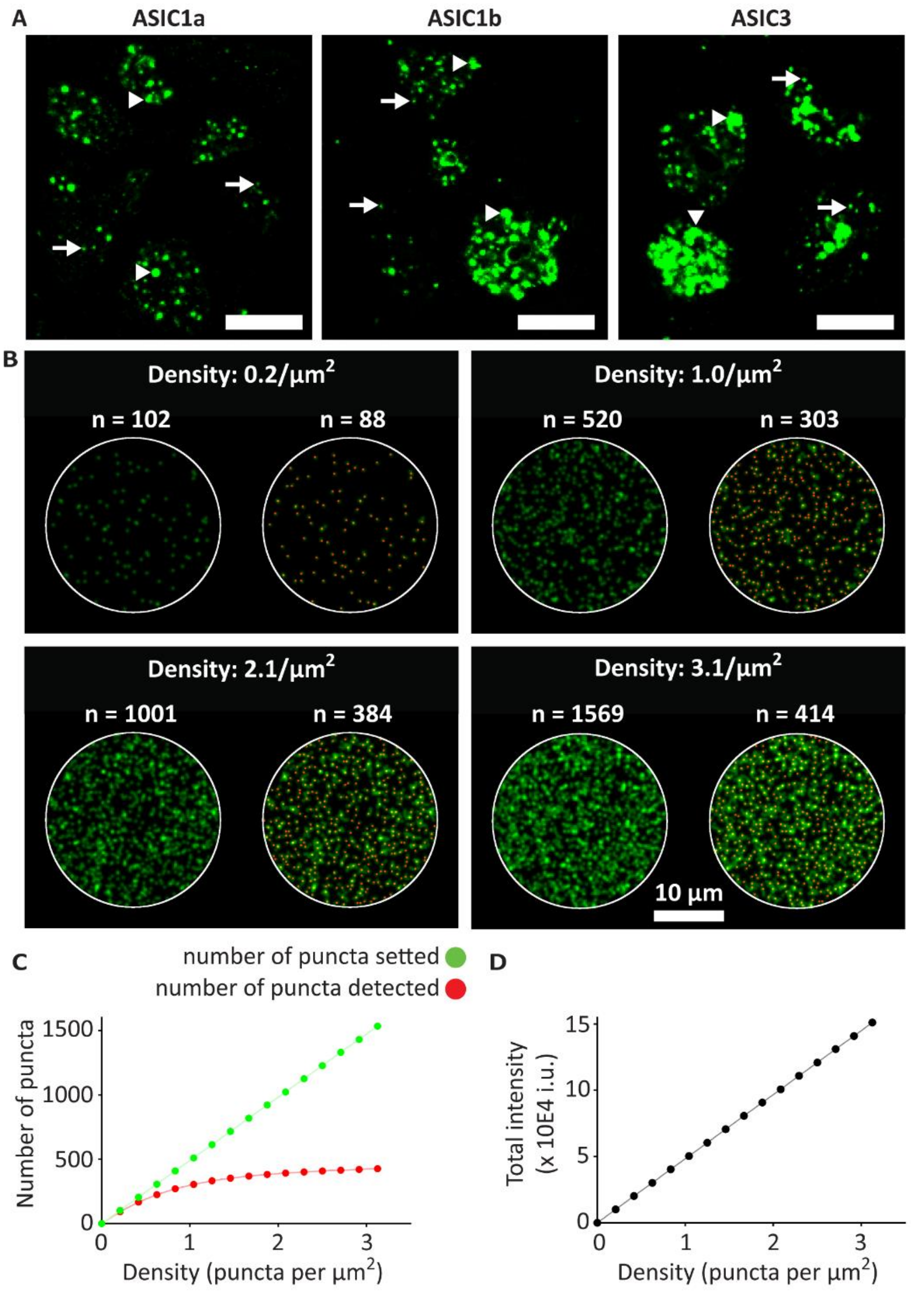

SUPPLEMENTARY FIGURE 3: Simulation of the relationship between fluorescent intensity and number of puncta. **(A)** The signal of ASICs revealed both puncta-like structure and small or large cluster-like structures. Arrows point towards single dots and arrowheads point towards clusters. Scale bar = 20 µm. **(B)** Simulated images of DRG neurons (25 µm diameter) containing various density of puncta. The left image corresponds to raw simulation (green) while the right image shows the detected puncta (red). **(C)** Plot showing the detected (red) *vs.* set (green) number of puncta. **(D)** Plot showing the estimate total intensity when density varied from 0.0001 to 3.1 puncta per µm^2. One thousand simulated images were generated and analyzed for each density. The error bars (SD) cannot be displayed because they are too small.

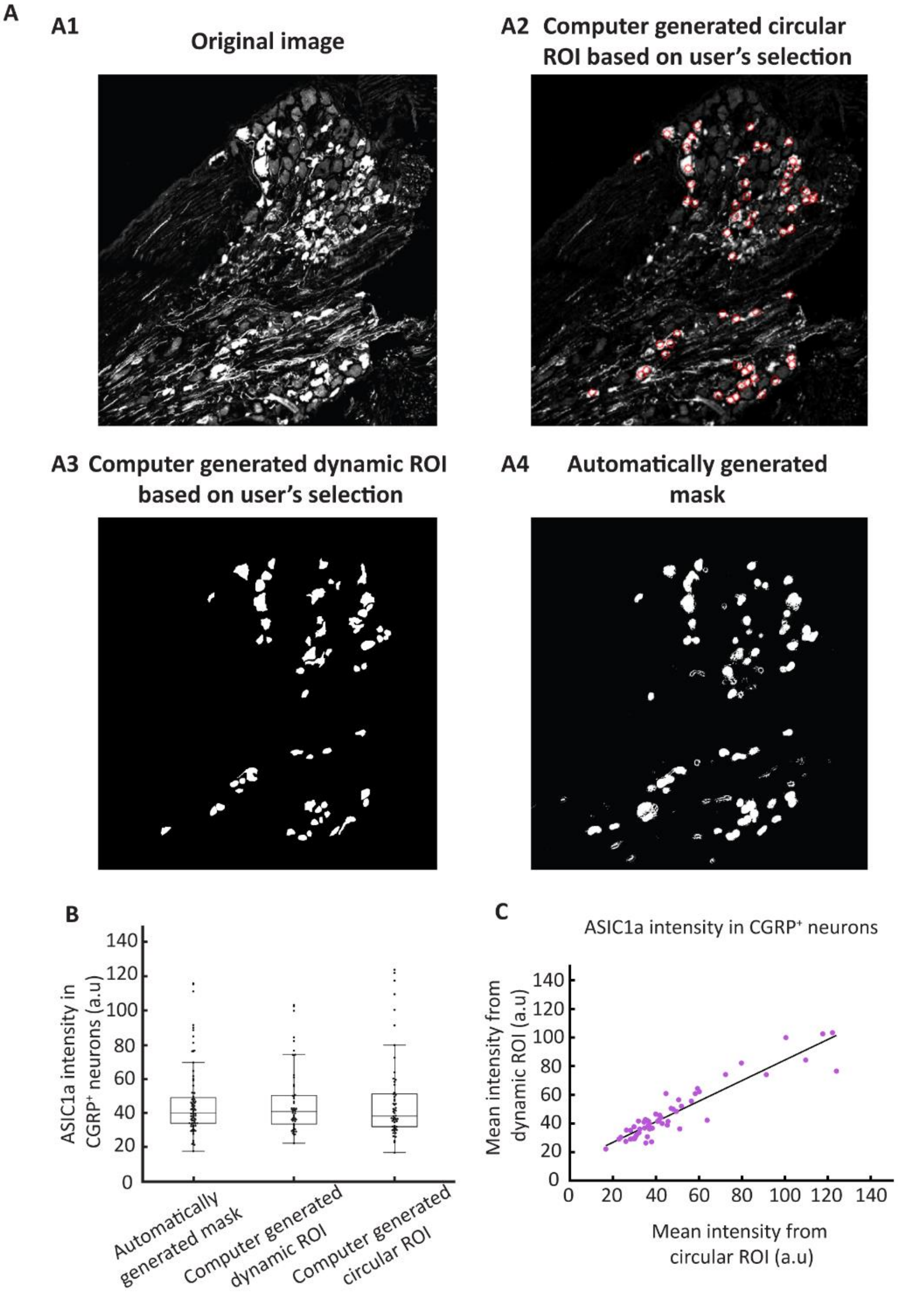

SUPPLEMENTARY FIGURE 4: Validation of the intensity analysis approach. **(A)** Example of the same image presenting CGRP$^+$ neurons segmented with three different approaches. Original image showing a DRG section with CGRP labeling (A1), red computer-generated circular ROIs on CGRP$^+$ neurons, based on experimenter's selection (A2), computer-generated dynamic ROIs covering the whole neuron, based on experimenter's selection (A3), fully automated mask covering the whole neuron generated by computer without any supervision (A4). **(B)** The mean intensity of ASIC1a in CGRP$^+$ neurons was measured with the three different approaches. No significant difference was found among them with Wilcoxon rank sum test. 77 ROIs for the automatically generated mask, 58 neurons for both computer generated circular and dynamic ROI approaches. Results are presented as MEAN ± SD. **(C)** High linear correlation between ASIC1a intensity derived from the computer-generated circular ROI and computer-generated dynamic ROI (R^2 = 0.86); 58 neurons for both approaches.
